# Effects of a Novel Consensus Bacterial 6-Phytase Variant on Growth Performance and Bone Ash of Broilers Fed Complex Diets Highly Deficient in Minerals, Digestible Amino Acids and Energy through 42 Days of Age

**DOI:** 10.3390/ani14111563

**Published:** 2024-05-25

**Authors:** Eric B. Sobotik, Gabrielle M. House, Austin M. Stiewert, Abiodun Bello, Yueming Dersjant-Li, Leon Marchal, Gregory S. Archer

**Affiliations:** 1 Poultry Science Department, Texas A&M University, College Station, TX 77843, USAhouseg@tamu.edu (G.M.H.);; 2 Danisco Animal Nutrition, IFF, 2342 BH Oegstgeest, The Netherlands; 3 Animal Nutrition Group, Wageningen University & Research, 2342 BH Oegstgeest, The Netherlands

**Keywords:** phytase, broiler, performance, full matrix

## Abstract

**Simple Summary:**

It is well-accepted by poultry nutritionists that phytase can contribute to increased P and Ca availability; however, despite increasing evidence reporting that there are extra-phosphoric effects of phytase, there is currently less acceptance of matrix values for digestible AA and energy. Nevertheless, phytase has been shown to improve ileal AA availability and energy utilization, and it therefore may be beneficial to assign AA and energy matrix values to phytase to not only capture the economic savings, but also to increase the magnitude of responses following phytase supplementation. Therefore, a study was conducted to evaluate the effect of increasing dose level of a novel consensus bacterial 6-phytase variant expressed in *Trichoderma reesei* in broilers fed a diet highly deficient in nutrients and energy. The hypothesis was that the novel consensus phytase variant would reduce the anti-nutritional effects of phytate, compensate for the reduction in minerals, dig AA, and energy in the basal negative control diets due to the capacity of improving the utilization of phytate-P, Ca, AA, and energy, resulting in improved performance and mineral utilization, which was confirmed.

**Abstract:**

An experiment was conducted to evaluate the effects of increasing the dose of a novel consensus bacterial 6-phytase variant expressed in *Trichoderma reesei* (PhyG) in broilers fed complex diets highly deficient in minerals, dig AA, and energy. Diets were a nutrient-adequate control (PC); a nutrient-reduced control (NC) formulated with a reduction in available P (avP) by 0.199%, Ca by 0.21%, crude protein by 0.72–1.03%, dig Lys by 0.064–0.084%, Na by 0.047%, and ME by 87.8 kcal/kg, respectively; and NC supplemented with PhyG at 500, 1000, and 2000 FTU/kg feed. BW was decreased and FCR increased in the NC vs. PC, while the PhyG treatments were similar to the PC. Carcass yield and bone ash were also maintained with PhyG supplementation. Phytase provided economic benefit on a feed cost per kg of weight basis for 1 to 35 d; the cost reductions equated to USD 0.006, 0.016, and 0.02/kg BWG at 500, 1000, and 2000 FTU/kg. In conclusion, this trial demonstrated that supplementation with a novel consensus phytase variant in diets highly deficient in minerals, dig AA, and energy maintained growth performance and provided economic benefit, with production benefits being maximized at inclusion levels of 2000 FTU/kg.

## 1. Introduction

The improvements in phosphorus (P) availability and digestibility that can be achieved with exogenous phytase are well-recognized for poultry [1,2]. It should be noted that the efficacy of phytase is not only limited to P digestibility. The current generation of phytase is more efficient, by increasing the availability and utilization of other minerals, amino acids (AAs), and energy, known as the extra-phosphoric effect [3,4,5]. Phytase contributions for increased P and Ca availability are generally well-accepted by poultry nutritionists. However, despite increasing evidence reporting the extra-phosphoric effects of phytase [3,4,5], there is currently less acceptance of matrix values for digestible AA and energy. Utilizing assigned AA and energy matrix values for phytase may lead to not only increased magnitude of response but also to increased economic savings as phytase has been shown to improve ileal AA availability and energy utilization.

Within the gastrointestinal tract (GIT) of poultry, phytate can bind to proteins, forming binary (phytate–protein) or ternary (phytate–mineral–protein) complexes [6], making phytate (myo-inositol hexaphosphate) poorly digested by poultry. The bioavailability of phytate-bound P is reduced when these complexes form. Furthermore, other minerals, proteins, and nutrients that are also bound to phytate [3,6] become less bioavailable as well. The binding of phytate with proteins can increase the rate of endogenous AA excretion, which increases mucin secretion and interferes with the intestinal uptake of AA via sodium (Na)-dependent transport systems [7,8]. To reduce the anti-nutritional effect of phytate on protein digestion, a phytase needs to be able to break down phytate more quickly in the acidic part of the GIT [5,9,10]. Next-generation phytase, such as a novel consensus bacterial 6-phytase variant, has a broad pH profile, has high relative activity at low pH in the foregut [11], and is capable of initiating phytate hydrolysis early enough to minimize the anti-nutritional effects of phytate. Given the increased pressure to reduce feed costs and maximize feed utilization, any reduction in dietary nutrients without loss of performance represents a potential cost savings for producers. 

It is well documented that the ingestion of phytate can have substantial adverse effects on endogenous secretion and energy utilization in poultry [7,12,13]. Phytate can form phytate–mineral complexes that may contribute to the formation of insoluble metallic soaps in the GIT, which limits lipid utilization [14]. Phytase supplementation can reduce the degree of soap formation and enhance the utilization of energy-derived lipids. In addition, phytate can increase the endogenous losses of Na in poultry [7]. Sodium deficiency can have a direct impact on the activity of Na^+^ K^+^-ATPase in the GIT that may compromise the Na-dependent transport mechanisms in the intestinal uptake of glucose [15,16]. Several studies have demonstrated that phytase inclusion substantially improved ileal Na digestibility coefficients [16,17,18]. The extra-phosphoric effects of phytase can offer additional value to producers due to feed cost savings by utilizing down-specification of energy and Na with no loss in production. 

The objective of this study was to evaluate the effect of increasing dose level of a novel consensus bacterial 6-phytase variant expressed in *Trichoderma reesei* in broilers fed a diet highly deficient in nutrients and energy. The working hypothesis was that the novel consensus phytase variant would reduce the anti-nutritional effects of phytate, compensate for the reduction in minerals, dig AA, and energy in the basal negative control diets due to the capacity of improving the utilization of phytate-P, Ca, AA, and energy of broilers fed complex diets through 42 days of age. 

## 2. Materials and Methods

### 2.1. Birds, Housing, and Experimental Design

All experimental protocols were approved by the Animal Care and Use Committee of Texas A&M University, USA (AUP #2018-0181). A total of 1560 Cobb 500-day-old broilers (straight-run) were obtained from a commercial hatchery and assigned to five dietary treatments, each containing 26 birds, and with twelve replicates per treatment, in a completely randomized block design (CRBD). Birds were weighed and randomly assigned to floor pens (1.22 × 1.83 m). Each pen was lined with new pine shavings. The pens had a surface area of 2.2 m^2^, resulting in an initial stocking density of 11.8 birds/m^2^ rising to 1 kg/m^2^ at 42 d. Birds were housed in an environmentally controlled tunnel ventilated house. Birds were housed at a density of 26 birds per pen at the end of the starter (1 to 10 d) and grower (11 to 21 d) phases. This decreased to 20 birds per pen at the end of the finisher 1 (22 to 35 d) and finisher 2 (36 to 42 d) phases following sampling of birds at the end of the grower phase. Feed and water were offered ad libitum throughout the trial.

### 2.2. Treatments, Diets, and Enzymes 

Birds were fed 4-phased diets in the form of a crumble during the starter phase and a pellet from the grower phase onwards. Five treatments were used, as follows: a positive control (PC) and one negative control (NC). The PC diet was formulated to meet nutritional requirements based on the Cobb guidelines. The NC diet contained 0.33% phytate-P and PC, respectively, reduced in ME by 87.8 kcal/kg, available P (avP) by 0.199%, Ca by 0.21%, crude protein by 0.72–1.03%, dig Lys by 0.064–0.084% (other AA down-spec; see Table 1), and Na by 0.047%. This very high nutrient/reduced energy is used to increase the response of phytase with increasing phytase dose from 500 to 2000 FTU/kg. Other diets included the NC, supplemented with a novel consensus bacterial 6-phytase variant in *Trichoderma ressei* (PhyG) at 500 (PhyG500), 1000 (PhyG1000), and 2000 (PhyG2000) FTU/kg. The ingredient and nutrient contents of the phased diets are presented in Table 1. Titanium dioxide was included at 0.5% of the diet, at the expense of corn, in the grower phase and used for the determination of ileal nutrient digestibility. All feed was pelleted at 24 °C.

### 2.3. Sampling Measurements

Body weight and feed intake (FI) were measured on day 0, 10, 21, 35, and 42, on a per-pen basis, and used to calculate average daily gain (ADG), average daily feed intake (ADFI), and mortality-corrected feed conversion ratio (FCR). On day 21 and 42, 6 birds per pen (3 males and 3 females) had both tibias collected, and the tibia ash was analyzed [19]. Ileal digesta were collected from the euthanized birds on day 21 and analyzed for apparent ileal digestibility (AID) [19]. On day 42, 5 birds per pen (3 males and 2 females) were weighed then euthanized by exsanguination and carcass component yields were measured using certified standard commercial processing procedures. Phytase activity was analyzed from representative feed samples within each dietary treatment (Table 2). For each dose level of phytase, the economic benefit was estimated based on feed cost in USD per kg of BWG (year 2020).

### 2.4. Chemical Analyses 

Phosphorus and Ca in feed and ileal digesta were analyzed by microwave digestion and Inductively Coupled Plasma-Optical Emission Spectrometry (OES) in accordance with method AOAC, 2011.14 [20]. Titanium dioxide concentration in feed and ileal digesta was analyzed using a modified protocol [21]. Following the procedures presented in Sobotik et al. [19] and described in brief here, dried samples were weighed and placed in an ashing oven at 450 °C. Samples were then titrated with sulfuric acid and boiled until dissolved and then further titrated with hydrogen peroxide and water. Finally, samples were analyzed for absorption using a spectrophotometer.

Phytase activities in the diets were determined by Danisco Animal Nutrition Laboratories (Brabrand, Denmark) utilizing a modified version of the 2001 AOAC method described in Engelen et al. [22]. 

### 2.5. Calculations 

Apparent ileal digestibility of P and Ca was calculated based on the following formula, using titanium dioxide as the inert marker: AID = 1 − [(Tid/Tii) × (Ni/Nd), where Tid is the titanium concentration in the diet, Tii is the titanium concentration in the ileal digesta, Ni is the nutrient (P or Ca) concentration in the ileal digesta, and Nd is the nutrient concentration in the diet. 

### 2.6. Statistical Analysis 

All data were subjected to an Analysis of Variance (ANOVA) to determine differences between treatments using a CRBD, using the Fit Model Platform of JMP 14.0 (JMP, SAS Institute Inc., Cary, NC, USA). Data were tested using Levene’s test for homogeneity of variance and the Shapiro–Wilk test for normality. Linear and quadratic responses were analyzed with increasing phytase dose from 0 (NC) to 2000 FTU/kg. Differences were considered statistically significant at *p* < 0.05. Tukey’s Honest Significant Differences test was used for post hoc separation of means. 

## 3. Results

### 3.1. Growth Performance 

Effects of treatment on growth performance per phase and cumulatively are presented in Table 3 and Table 4. Treatment affected most response measures during all growth phases (starter, grower, finisher 1, finisher 2, and overall; *p* < 0.05). Compared to PC, birds fed NC diet exhibited reduced BW at 42 d (2588 vs. 2988 g), increased overall FCR (1.80 vs. 1.60), and reduced ADG (60.4 vs. 69.9 g/bird/day) and ADFI (103 vs. 114 g/bird/day) from 1 to 42 d (*p* < 0.05). Supplementation with phytase, at any dose level, allowed the birds to overcome the adverse effects of the highly nutrient- and energy-deficient NC diet with improved BW, FCR, ADG, and ADFI during all phases vs. NC (*p* < 0.05) and such that they were equivalent to the PC. The application of PhyG at 500, 1000, and 2000 FTU/kg produced birds with a mean BW at 42 d (2880, 2920, and 2947 g vs. 2988 in PC), overall FCR (1.71, 1.67, 1.66 vs. 1.63 in PC), and ADG (67.3, 68.3, 69.0 g/bird/day vs. 69.9 in PC) and ADFI (114, 114, 114 g/bird/day vs. 114 in PC) from 1 to 42 d (*p* > 0.05). Increasing phytase dose from 0 (NC) to 2000 FTU/kg resulted in linear and quadratic response in BW, FCR, ADG, and ADFI in most phases (*p* < 0.05). 

### 3.2. Tibia and Carcass Characteristics 

Effects of treatment on tibia ash and breaking strength are presented in Table 5. Compared to PC, birds fed NC diet exhibited reduced tibia ash at day 21 and day 42 (−7.2 and −5.1 percentage points, respectively; *p* < 0.05). Compared to PC, birds fed NC diet also exhibited reduced tibia breaking strength at day 21 and day 42 (−8.7 and −7.4 kg, respectively; *p* < 0.05). Supplementation with phytase at any dose level alleviated the adverse effects of the highly nutrient- and energy-deficient NC diet (*p* < 0.05) and completely restored tibia ash at both day 21 and day 42 such that they were equivalent with PC. Similar results for breaking strength were also observed with phytase supplementation at day 21 and day 42. At both time points, the greatest tibia ash and breaking strength were observed with phytase at 2000 FTU/kg. Increasing phytase dose from 0 (NC) to 2000 FTU/kg resulted in linear and quadratic response in tibia ash and breaking strength at both day 21 and day 42.

The effects of treatment on carcass component weights and yields are presented in Table 6. Compared to PC, birds fed NC diet exhibited markedly reduced (*p* < 0.05) eviscerated carcass weight (2127 vs. 2466 g), breast weight (505 vs. 619 g), tender weight (107 vs. 130 g), leg quarter weight (690 vs. 775 g), and fat pad weight (21 vs. 30 g). Compared to PC, birds fed NC also exhibited reduced (*p* < 0.05) carcass yield (76.10 vs. 77.82%), breast yield (23.69 vs. 25.07%), and fat pad yield (1.00 vs. 1.24%). Supplementation with phytase, at any dose level, allowed the birds to overcome the adverse effects of the highly nutrient- and energy-deficient NC diet with improved carcass characteristics and yields such that they were equivalent to the PC. The application of PhyG at 500, 1000, and 2000 FTU/kg produced birds with a mean carcass yield of (77.58, 77.86, and 77.94% vs. 77.82 in PC) and breast yield of (24.34, 24.67, and 25.10% vs. 25.07 in PC). Increasing phytase dose from 0 (NC) to 2000 FTU/kg resulted in a linear and quadratic response in eviscerated carcass weight, breast weight, tender weight, leg quarter weight, fat pad weight, carcass yield, breast yield, and fat pad yield. 

### 3.3. Nutrient Digestibility 

The effects of nutrient digestibility are presented in Table 7. Compared to PC, birds fed NC diet exhibited reduced AID of P and Ca (−11.4 and −14.3 percentage points, respectively; *p* < 0.05). Supplementation with phytase at any dose level improved P and Ca digestibility. Increasing phytase dose from 0 (NC) to 2000 FTU/kg resulted in a linear and quadratic increase in AID of both P and Ca. 

### 3.4. Economic Analysis 

The effects of treatment on economic benefit are presented in Table 8. On a feed costs per kg of weight basis for 1 to 35 d, the cost reductions equated to USD 0.006/kg BWG when phytase was dosed at 500 FTU/kg, USD 0.016/kg BWG at 1000 FTU/kg, and USD 0.02/kg BWG at 2000 FTU/kg. For 1 to 42 d, the cost reductions equated to USD 0.015/kg BWG when phytase was dosed at 500 FTU/kg, USD 0.022/kg BWG, and USD 0.029/kg BWG at 2000 FTU/kg. 

## 4. Discussion

Results from the present study have demonstrated a positive dose-dependent effect of the novel consensus phytase variant on growth performance of broilers fed complex diets highly deficient in minerals, dig AA, and energy. The impaired growth performance of birds fed the NC diet compared with the nutritionally adequate PC diet gave a clear indication of its nutritional inadequacy. These results were expected given the high reductions in nutrients and energy and suggest that one or more of the down-specified nutrients in the NC diet were limiting for growth. Compared with PC, birds fed NC diets reduced ADG and ADFI by 13.6 and 9.5%, respectively. The final BW was nearly 400 g lower in NC compared with PC, confirming that the nutrients and energy were highly deficient. These findings are in agreement with previous studies which have shown that dietary reductions in P, Ca, AA, and ME significantly reduced broiler growth performance compared with a nutritionally adequate diet [22,23,24]. The reductions in growth performance due to nutrient deficiency were reversed with the inclusion of phytase. In the current study, phytase supplementation, at each dose level, restored BW, maintained ADG and FCR, and improved ADFI to similar levels to the PC during all phases as well as cumulatively and overall (1 to 42 d). Whilst the responses in performance at all phytase dose levels were statistically equivalent to the PC, there was a clear dose–response effect observed, as evidenced by a linear and quadratic increase in most performance response measures with increasing phytase dose. Several recent studies have reported similar dose–response effects of phytase supplementation (0 to 2000 FTU/kg) on broiler growth performance [5,25,26]. The improvements in growth performance can be attributed to increases in nutrient availability as phytase dose was increased. This is confirmed by Kiarie et al. [26] in which a *Buttiauxella* phytase (0, 250, 500, 750, 1000, and 2000 FTU/kg) improved growth performance and utilization of nutrients in broilers in a dose-dependent manner. In that study, the benefits on AA and energy were maximized at higher doses of phytase. Dersjant-Li et al. [25] observed that *Buttiauxella* phytase at 500 and 1000 FTU/kg compensated for the reduction in nutrients (avP, Ca, Na, and AA) and energy (the matrix was applied at respective dose level) and maintained growth performance of broilers through 42 days of age. These results, along with those from the current study, indicate the high phosphoric and extra-phosphoric efficacy of the next-generation phytase to compensate for the high nutrient and energy down-specification applied and recover the performance of birds compared with a nutrient-adequate diet. There was a clear dose–response effect, as evidenced by a linear and quadratic increase in performance with increasing phytase dose. It can be expected that the action of PhyG at 2000 FTU/kg in degrading phytate maximized the availability of P, Ca, dig AA, and energy, which compensated for the nutrient reductions in the diet. However, increased FCR was observed during the grower phase, which might be related to the very high down-specification (88 kcal/kg) of energy. The ME starting level (ME Poultry) is lower than breeder recommendations, which may explain the numerically increased FCR. This study was with complex diets based on corn, soybean meal, wheat, rapeseed meal, rice bran, and oat hulls containing high level of phytate and NSP (non-starch polysaccharide), without xylanase in the background. Starch represents the single largest energy-yielding component of poultry diets. It has been suggested that phytate may interfere with starch digestion by directly binding with starch via hydrogen bonds or phosphate linkages [27]. Moreover, phytate may be involved in the formation of insoluble metallic soaps in the gut lumen of poultry, which are major constraints on energy utilization [28]. Similarly, NSP may exhibit anti-nutritive activity when present at high levels in broiler diets. The high fiber content in the experimental diets may result in increased water holding capacity, increased digesta retention time, and reduced feed intake [29]. Another possible cause could be the source of dietary Ca. This study utilized a highly soluble limestone, which can have negative impact. Limestone is the dominant source of Ca in poultry diets, and has an extremely high acid binding capacity, and will tend to increase digesta pH along the gut [30]. Gut pH will directly influence exogenous phytase activity depending on the pH activity of the specific enzyme [31]. In addition, limestone has the potential to increase the formation of insoluble Ca–phytate complexes by increasing both pH and Ca–phytate molar ratios [31]. The results from the current study are not directly comparable to other studies, due to different experimental settings: phytate, NSP, limestone solubility, and ME starting level. In practice, when applying phytase matrix values, these findings suggest that in diets that are formulated based on breeder recommendations and supplemented with phytase, applying an ME matrix should be linked to dietary settings, including starting ME level. 

The anti-nutritional effects of phytate on protein and AA digestibility are well-reviewed [3,6,32]. Based on the available literature, Selle et al. [6] has proposed three possible mechanisms by which phytate can reduce protein and AA absorption: (1) via binding to AA during the gastric phase of digestion, forming binary or ternary protein–phytate complexes that are resistant to digestion by pepsin, (2) via increased excretion of endogenous AA in high-phytate diets as a result of increased mucin secretion, and (3) via interference with starch, glucose, and AA absorption from the gut lumen by compromising the Na+-dependent transport systems. Moreover, Yu et al. [33] reported that IP6 has a much greater binding capacity to protein compared to lower esters (IP5, IP4, IP3) and thus has a greater negative impact on protein digestion. To reduce the negative effects of phytate on protein digestion, a phytase needs to be able to break down phytate quickly in the acidic conditions of the upper gastrointestinal tract (GIT; proventriculus and gizzard), reducing the formation of protein–phytate complexes, and improving the digestibility of AA as a result of an extra-phosphoric effect. In the current study, birds fed the NC diet reduced eviscerated carcass weight (−339 g), breast weight (−114 g), tender weight (−23 g), leg weight (−85 g), fat pad weight (−9 g), carcass yield (2.3%), and breast yield (5.8%) vs. PC. These reductions are likely the result of a diet highly deficient in nutrients (e.g., dig AA) and energy. Supplementation with phytase, at any dose level, allowed the birds to overcome the adverse effects of the nutrient- and energy-deficient NC diet to maintain carcass weights and yields such that they were equivalent to the PC. Increasing phytase dose from 0 (NC) to 2000 FTU/kg resulted in a linear and quadratic response in eviscerated carcass weight, breast weight, tender weight, leg quarter weight, fat pad weight, carcass yield, breast yield, and fat pad yield. Thus, in addition to the beneficial effects on P availability, the novel consensus phytase variant can improve the digestibility of AA, demonstrating its extra-phosphoric effects. This compares well with existing literature relating to increasing levels of phytase in diets containing variable levels of AA on processing yields. In a study conducted by Smith et al. [34], phytase level positively impacted carcass component yield, as increasing phytase dose from 500 FTU/kg to 1500 FTU/kg increased breast yield in reduced-AA diets. Numerous studies have reported varying degrees of improvement in protein and AA digestibility in response to 500 to 4000 FTU/kg phytase from a range of sources in both wheat- and corn-based diets [5,22,30,34]. Exogenous phytase can improve AA digestibility by several modes of action: (1) prevention of binary protein–phytate complex formation in the upper GIT at less than the isoelectric point of protein and ternary protein–phytate complex formation at more than the isoelectric point of protein, (2) reduction in mucin-associated endogenous AA flows, and (3) improved intestinal uptakes of amino acids by Na-dependent transporters via facilitated Na-K-ATPase pump activity [30]. The upper GIT, in which pH may be as low as 2.5 to 3.0, is the critical site for phytase to improve AA digestibility [31]. It seems likely the effects on carcass characteristics in the current study were mediated by the degradation of dietary phytate by PhyG in the upper GIT, leading to a reduced presence of phytate and its adverse effect on protein utilization. This is in agreement with Christensen et al. [11], who demonstrated that PhyG exhibited high relative activity over a wide pH range (pH 2.0 to 5.5), with a maximum activity at pH 4.0 (233% of activity at pH 5.5, respectively), and substantial activity retained at pH 1.5 (89% of activity at pH 5.5). More specifically, the novel consensus phytase variant is more active in the upper part of the digestive tract, where the environment is more acidic, and can rapidly degrade phytate to more innocuous esters and reduce the formation of protein–phytate complexes. These results, along with those from the current study and Dersjant-Li et al. [35] indicate the high phosphoric and extra-phosphoric efficacy of the next-generation phytase to compensate for the high nutrient and energy down-specification applied and maintain carcass characteristics equivalent to those of a nutrient-adequate diet. There was a clear dose–response effect, as evidenced by a linear and quadratic increase in carcass characteristics with increasing phytase dose. It can be hypothesized that the action of PhyG at 2000 FTU/kg in degrading phytate maximized the availability of protein and AA vs. PC.

Bone quality measurements such as tibia ash and breaking strength are good indicators of bone mineralization and have been commonly used for evaluating phytase efficacy on mineralization and deposition in broilers [3]. In the current study, bone mineralization was adversely affected in birds fed the NC diet, which may have resulted from insufficient levels of P and Ca available for bone development. Supplementation with phytase, at any dose level, allowed the birds to overcome the adverse effect of the mineral reductions and completely restored tibia ash and breaking strength to the same levels as the PC at each time point (21 and 42 d). Increasing phytase dose from 0 (NC) to 2000 FTU/kg resulted in a linear and quadratic response in tibia ash and breaking strength. Based on these results, it can be suggested the addition of phytase in the current study improved the availability of P and Ca, resulting in greater digestibility of these minerals and higher concentrations of substrate for bone mineral deposition. This compares well with existing literature relating to dose–response effects of phytase on broilers within a range of 0 to 4000 FTU/kg [5,34,36]. In a study conducted by Dersjant-Li et al. [36], tibia ash in all three phytase treatments, 250, 500, and 1000 FTU/kg, was equivalent to the nutrient-adequate diet; however, the response was numerically highest with phytase at 1000 FTU/kg. Similar observations were made by Babatunde et al. [37], with a quadratic response in tibia ash (37.2 to 47.9%) and tibia ash weight (298 to 453 mg/bone) with increasing phytase dose from 0 to 4000 FTU/kg for a *Buttiauxella* phytase. These results, along with those from the current study, indicate that phytase maintained bone quality in a diet highly deficient in nutrients and energy. This validates the efficacy of the next-generation phytase to improve mineral utilization and fortify skeletal structure. There was a clear dose–response effect, as evidenced by a linear and quadratic increase in bone quality with increasing phytase dose. It can be expected that the action of PhyG at 2000 FTU/kg in degrading phytate maximized the availability of P and Ca vs. PC

The impact of phytase in nutrient digestibility has previously been evaluated, with consistent improvements in nutrient utilization being reported [2,38]. In the current study, birds fed the NC diet highly deficient in nutrients and energy had lower AID of P (−11.4 percentage points) and Ca (−14.3 percentage points) as compared with the nutrient-adequate PC, respectively. As expected, phytase supplementation at any each dose level improved apparent ileal digestibility of P and Ca. Increasing phytase dose from 0 (NC) to 2000 FTU/kg resulted in a linear and quadratic response in nutrient digestibility. These results are not surprising, as greater improvements in ileal digestibility were observed as phytase dose increased, indicating that P and Ca digestibility may be attributed to the increased liberation of phytate-P and removal of its anti-national effects via phytase inclusion [16]. These findings confirm the efficacy of the next-generation phytase in releasing phytate-P and improving availability of Ca. Numerous studies have exhibited improvements in P and Ca availability when supplementing with phytase in nutrient-reduced diets [34,38,39]. However, comparison of the current ileal digestibility data with those from other studies evaluating *Buttiauxella*-derived phytases is difficult because of differences in the concentrations of phytate and phytase. This study was conducted with a high level of phytate-P (0.33%) and limestone with high solubility. In addition, experimental settings were also different from those presented in the literature. Therefore, a direct comparison of ileal digestibility with the existing literature is not appropriate. These results, along with those from the current study, suggest that phytase at higher dose levels leads to greater destruction of phytate, thus releasing additional phosphate molecules for absorption, and reduces the binding of Ca to phytate. There was a clear dose–response effect, as evidenced by a linear and quadratic increase in nutrient digestibility with increasing phytase dose. It can be expected that the action of PhyG at 2000 FTU/kg in degrading phytate maximized the availability of P and Ca. 

Economic analysis was conducted to evaluate the extent of any feed cost reduction that can be achieved by the novel consensus phytase variant when applied in conjunction with a full nutrient and energy matrix in complex broiler diets. In terms of how each dose level of phytase affected economic benefit, which was estimated based on feed cost in USD per kg of BWG, from 1 to 35 d, phytase supplementation of the NC diet with phytase at 500, 1000, and 2000 FTU/kg resulted in an average cost savings of USD 0.006, 0.016, and 0.02/kg BWG. Similarly, from 1 to 42 d, phytase supplementation at 500, 1000, and 2000 FTU/kg resulted in an average cost savings of USD 0.015, 0.023, and 0.029/kg BWG. Based on these estimates, phytase supplementation, at each dose level, improved economic benefit relative to the nutrient-adequate PC. However, phytase at a dose- level of 2000 FTU/kg was shown to be more cost-effective in lowering production cost relative to phytase at 1000 to 500 FTU/kg. 

## 5. Conclusions

The overall findings of this study demonstrated that the novel consensus bacterial 6-phytase variant fully compensated for the high reduction in nutrients and energy at each dose level and maintained growth performance, carcass characteristics, bone quality, and nutrient digestibility of broilers, leading to reduced feed cost and production benefit. Furthermore, production and economic benefits were maximized with phytase at inclusion levels of 2000 FTU/kg. The results from the current study indicate the high extra-phosphoric efficacy of the next-generation phytase on top of the phosphoric efficacy in complex diets fed to broilers through 42 days of age.

## Figures and Tables

**Table 1:** Ingredient and calculated (Concept 5 feed formulation software, Version 5) and analyzed (Midwest Labs, USA) nutrient content (%, as fed basis) of the basal PC and NC diet, by phase.

	Starter (d 1 to 10)		Grower (d 11 to 21)		Finisher 1 (d 22 to 35)		Finisher 2 (d 36 to 42)	
Ingredient, %	PC	NC *	PC	NC *	PC	NC *	PC	NC *
Corn	38.01	34.42	38.42	34.14	38.80	34.92	39.85	34.94
Soybean meal	29.85	26.42	25.68	22.62	20.75	18.26	19.02	16.08
Wheat, grain	19.01	20.21	20.06	22.05	22.97	24.93	23.10	26.52
Rapeseed meal	4.50	6.00	5.18	6.65	6.00	7.00	6.50	8.00
Rice bran	3.00	4.66	4.00	5.32	5.00	5.80	5.00	5.81
Oat hulls	0.86	4.98	1.00	5.00	1.00	5.01	1.00	4.69
Soy oil	0.67	0.59	1.95	1.87	2.20	2.15	2.43	2.25
Mono-calcium phosphate	1.43	0.41	1.19	0.16	1.02	-	1.03	-
Limestone	1.22	1.12	1.17	1.06	0.96	0.85	0.96	0.85
Salt	0.38	0.25	0.37	0.25	0.37	0.24	0.37	0.24
DL-Methionine	0.33	0.29	0.27	0.23	0.25	0.21	0.16	0.11
L-Lysine HCL	0.30	0.25	0.31	0.28	0.30	0.28	0.22	0.19
L-Threonine	0.15	0.11	0.10	0.07	0.08	0.05	0.05	0.01
Vit and Min Premix ^1^	0.30	0.30	0.30	0.30	0.30	0.30	0.30	0.30
**Calculated nutrients, %**								
ME, kcal/kg	2950	2862	3050	2962	3100	3012	3120	3032
Calcium	0.89	0.68	0.82	0.61	0.70	0.49	0.70	0.49
Tot. phosphorus	0.83	0.62	0.78	0.56	0.74	0.51	0.74	0.51
Av phosphorus	0.43	0.23	0.38	0.18	0.34	0.14	0.34	0.14
Sodium	0.17	0.12	0.17	0.12	0.17	0.12	0.17	0.12
Crude protein	21.81	20.78	20.35	19.50	18.86	18.06	18.18	17.46
dig Lysine	1.22	1.14	1.12	1.05	1.02	0.96	0.92	0.86
dig Methionine	0.60	0.54	0.56	0.51	0.52	0.47	0.43	0.37
dig TSAA	0.91	0.84	0.85	0.79	0.80	0.74	0.70	0.64
dig Threonine	0.83	0.76	0.73	0.67	0.66	0.61	0.61	0.56
dig Tryptophan	0.22	0.21	0.21	0.20	0.19	0.18	0.18	0.18
dig Arginine	1.28	1.21	1.18	1.12	1.07	1.02	1.03	0.98
dig Isoleucine	0.80	0.76	0.74	0.71	0.68	0.65	0.65	0.62
dig Leucine	1.53	1.44	1.43	1.35	1.32	1.26	1.29	1.22
dig Valine	0.89	0.86	0.84	0.81	0.78	0.75	0.76	0.73
**Analyzed nutrients**								
Crude protein	21.4	19.7	21.5	18.90	19.8	18.90	18.0	17.50
Calcium	1.05	0.63	0.81	0.69	0.75	0.54	0.72	0.62
Tot. phosphorus	0.82	0.58	0.74	0.53	0.70	0.49	0.72	0.50

^1^ Vitamin premix contained 8818 IU vitamin A, 3086 IU vitamin D3, 37 IU vitamin E, 0.0132 mg B12, 4.676 mg riboflavin, 36.74 mg niacin, 16.17 mg d-pantothenic acid, 382.14 mg choline, 1.18 mg menadione, 1.4 mg folic acid, 5.74 mg pyridoxine, 2.35 mg thiamine and 0.44 mg biotin per kg diet and trace mineral premix contained 149.6 mg manganese, 125.1 mg zinc, 16.5 mg iron, 1.7 mg copper, 1.05 mg iodine, 0.25 mg selenium, a minimum of 6.27 mg calcium, and a maximum of 8.69 mg calcium per kg of diet. The carrier was calcium carbonate and the premix contained less than 1% mineral oil. * with reduction vs. PC of 87.8 kcal/kg ME, 0.199% avP, 0.21% Ca, 0.72–1.03% crude protein, 0.064–0.084% dig Lys, and 0.047% Na.

**Table 2:** Analyzed phytase (FTU/kg) values of diets, by phase.

	Starter (d 1 to 10)	Grower (d 11 to 21)	Finisher 1 (d 22 to 35)	Finisher 2 (d 36 to 42)
PC	273	534	613	585
NC	323	331	362	394
NC+PhyG500	696	867	870	833
NC+PhyG1000	1061	1267	1034	1465
NC+PhyG2000	2165	2462	1959	2267

**Table 3:** Effect of full matrix application of a novel consensus phytase variant, on growth performance, by phase.

	Body Weight, g	Average Daily BW Gain, g	Average Daily Feed Intake, g	FCR
Starter, d 1–10 and BW at d 10				
PC	283 ^a^	23.9 ^a^	26.6 ^ab^	1.10 ^a^
NC	265 ^b^	22.0 ^b^	25.6 ^b^	1.16 ^b^
NC+PhyG500	280 ^a^	23.5 ^a^	27.0 ^a^	1.15 ^ab^
NC+PhyG1000	282 ^a^	23.7 ^a^	27.3 ^a^	1.15 ^ab^
NC+PhyG2000	288 ^a^	27.8 ^a^	27.8 ^a^	1.14 ^ab^
Pooled SEM	1.363	0.138	0.164	0.005
*p*-value	<0.001	<0.001	<0.001	0.040
*P-linear*	<0.001	<0.001	<0.001	0.259
*P-quadratic*	0.003	0.004	0.031	0.757
Grower, d 11–21 and BW at d 21				
PC	1008 ^a^	66.2 ^a^	91.9 ^a^	1.40 ^a^
NC	831 ^b^	51.8 ^b^	82.3 ^b^	1.59 ^c^
NC+PhyG500	981 ^a^	64.1 ^a^	95.4 ^a^	1.49 ^b^
NC+PhyG1000	995 ^a^	65.2 ^a^	94.5 ^a^	1.45 ^b^
NC+PhyG2000	992 ^a^	64.3 ^a^	93.2 ^a^	1.45 ^b^
Pooled SEM	9.724	0.806	0.724	0.011
*p*-value	<0.001	<0.001	<0.001	<0.001
*P-linear*	<0.001	<0.001	<0.001	<0.001
*P-quadratic*	<0.001	<0.001	<0.001	<0.001
Finisher 1, d 22–35 and BW at d 35				
PC	2272 ^a^	98.7 ^a^	164.7 ^a^	1.70 ^a^
NC	1969 ^b^	89.6 ^b^	148.0 ^b^	1.66 ^a^
NC+PhyG500	2200 ^a^	95.4 ^ab^	167.0 ^a^	1.75 ^b^
NC+PhyG1000	2237 ^a^	97.1 ^a^	167.0 ^a^	1.73 ^ab^
NC+PhyG2000	2246 ^a^	98.5 ^a^	168.0 ^a^	1.71 ^ab^
Pooled SEM	19.376	0.910	1.346	0.009
*p*-value	<0.001	0.006	<0.001	0.004
*P-linear*	<0.001	0.002	<0.001	0.382
*P-quadratic*	<0.001	0.078	<0.001	0.006
Finisher 2, d 36–42 and BW at d 42				
PC	2988 ^a^	104.3	220.7	2.20
NC	2588 ^b^	94.4	206.0	2.20
NC+PhyG500	2880 ^a^	103.0	218.0	2.14
NC+PhyG1000	2920 ^a^	101.0	214.0	2.16
NC+PhyG2000	2947 ^a^	106.0	224.0	2.17
Pooled SEM	31.966	2.668	2.481	0.047
*p*-value	0.002	0.913	0.350	0.953
*P-linear*	0.001	0.447	0.100	0.729
*P-quadratic*	0.049	0.922	0.809	0.878

All growth performance parameters (ADG, ADFI, and FCR) are corrected for mortality. ^a–c^ Means in the same row with no common superscripts are significantly different at a probability level of *p* < 0.05. SEM, pooled standard error of the mean.

**Table 4:** Effect of full matrix application of a novel consensus phytase variant, on growth performance, cumulatively.

	Average Daily BW Gain, g	Average Daily Feed Intake, g	FCR	Mortality, %
d 1–21				
PC	45.9 ^a^	60.6 ^a^	1.30 ^a^	2.60
NC	37.5 ^b^	55.1 ^a^	1.47 ^c^	3.50
NC+PhyG500	44.6 ^a^	62.4 ^a^	1.40 ^b^	3.20
NC+PhyG1000	45.2 ^a^	62.2 ^a^	1.38 ^b^	2.90
NC+PhyG2000	45.1 ^a^	61.8 ^a^	1.37 ^b^	3.20
Pooled SEM	0.460	0.407	0.008	0.396
*p*-value	<0.001	<0.001	<0.001	0.957
*P-linear*	<0.001	<0.001	<0.001	0.806
*P-quadratic*	<0.001	<0.001	<0.001	0.663
d 1–35				
PC	63.5 ^a^	95.2 ^a^	1.50 ^a^	2.90
NC	54.8 ^b^	86.2 ^b^	1.57 ^bc^	4.80
NC+PhyG500	61.4 ^a^	97.1 ^a^	1.58 ^c^	4.20
NC+PhyG1000	62.5 ^a^	97.2 ^a^	1.56 ^bc^	3.50
NC+PhyG2000	62.8 ^a^	96.8 ^a^	1.54 ^b^	4.20
Pooled SEM	0.501	0.664	0.005	0.471
*p*-value	<0.001	<0.001	<0.001	0.761
*p-linear*	<0.001	<0.001	0.007	0.690
*p-quadratic*	<0.001	<0.001	0.659	0.479
d 1–42				
PC	69.9 ^a^	113.8 ^a^	1.60 ^a^	4.20
NC	60.4 ^b^	103.0 ^b^	1.71 ^b^	5.80
NC+PhyG500	67.3 ^a^	114.0 ^a^	1.70 ^b^	3.80
NC+PhyG1000	68.3 ^a^	114.0 ^a^	1.67 ^ab^	3.80
NC+PhyG2000	69.0 ^a^	115.0 ^a^	1.67 ^ab^	5.90
Pooled SEM	0.686	0.836	0.007	0.528
*p*-value	<0.001	<0.001	<0.001	0.595
*P-linear*	0.001	<0.001	0.130	0.914
*P-quadratic*	0.010	<0.001	0.969	0.136

All growth performance parameters (ADG, ADFI, and FCR) are corrected for mortality; ^a–c^ Means in the same row with no common superscripts are significantly different at a probability level of *p* < 0.05. SEM, pooled standard error of the mean. FCRc, body weight-corrected FCR, calculated by correction of FCR values by 3 points per 100 g of BW difference from the PC.

**Table 5:** Effect of full matrix application of a novel consensus phytase variant, on tibia ash and breaking strength.

	Tibia Ash,%		Tibia Breaking Strength, kgF	
	d21	d42	d21	d42
PC	52.62 ^a^	53.35 ^a^	18.44 ^a^	33.41 ^a^
NC	45.39 ^c^	48.30 ^c^	9.76 ^b^	26.02 ^b^
NC+PhyG500	50.50 ^b^	52.03 ^b^	17.25 ^a^	32.74 ^a^
NC+PhyG1000	51.41 ^ab^	52.54 ^ab^	17.58 ^a^	33.02 ^a^
NC+PhyG2000	51.83 ^a^	53.22 ^ab^	18.05 ^a^	33.63 ^a^
Pooled SEM	0.359	0.280	0.478	0.459
*p*-value	<0.001	<0.001	<0.001	<0.001
*P-linear*	<0.001	<0.001	<0.001	<0.001
*P-quadratic*	<0.001	<0.001	<0.001	<0.001

^a–c^ Means in the same row with no common superscripts are significantly different at a probability level of *p* < 0.05. SEM, pooled standard error of the mean.

**Table 6:** Effect of full matrix application of a novel consensus phytase variant, on carcass weights and yields.

	Live BW, g	BW without Giblets, g	Breast wt, g	Tender wt, g	Leg wt, g	Fat Pad wt, kg
PC	3170.68 ^a^	2465.61 ^a^	619.22 ^a^	130.17 ^a^	774.83 ^a^	30.05 ^a^
NC	2794.78 ^b^	2127.12 ^b^	504.78 ^b^	107.31 ^b^	690.31 ^b^	21.18 ^b^
NC+PhyG500	3078.70 ^a^	2385.48 ^a^	582.34 ^a^	124.37 ^a^	766.82 ^a^	28.14 ^a^
NC2+PhyG1000	3101.38 ^a^	2411.86 ^a^	596.95 ^a^	126.06 ^a^	775.49 ^a^	29.19 ^a^
NC+PhyG2000	3130.83 ^a^	2438.78 ^a^	614.44 ^a^	126.81 ^a^	786.47 ^a^	30.23 ^a^
Pooled SEM	0.021	0.017	0.006	0.001	0.006	0.001
*p*-value	<0.001	<0.001	<0.001	<0.001	<0.001	<0.001
*P-linear*	<0.001	<0.001	<0.001	<0.001	<0.001	<0.001
*P-quadratic*	<0.001	<0.001	0.001	<0.001	0.001	0.003
		Carcass yield, %	Breast yield, %	Tender yield, %	Leg yield, %	Fat pad yield, %
PC		77.82 ^a^	25.07 ^a^	5.30	31.43	1.24 ^a^
NC		76.10 ^b^	23.69 ^b^	5.06	32.46	1.00 ^b^
NC+PhyG500		77.50 ^a^	24.39 ^ab^	5.25	32.12	1.20 ^ab^
NC2+PhyG1000		77.83 ^a^	24.68 ^ab^	5.25	32.17	1.22 ^ab^
NC+PhyG2000		77.94 ^a^	25.10 ^a^	5.23	32.26	1.25 ^a^
Pooled SEM		0.136	0.126	0.030	0.115	0.026
*p*-value		<0.001	0.002	0.091	0.055	0.010
*P-linear*		<0.001	<0.001	0.165	0.776	0.003
*P-quadratic*		0.004	0.319	0.087	0.325	0.070

^a,b^ Means in the same row with no common superscripts are significantly different at a probability level of *p* < 0.05. SEM, pooled standard error of the mean.

**Table 7:** Effect of a full matrix application of a novel consensus phytase variant, on apparent ileal digestibility (AID, %) of Ca and P in broilers 21 days of age.

	PC	NC	NC+PhyG500	NC+PhyG1000	NC+PhyG2000	Pooled SEM	*p*-Value	*P-Linear*	*P-Quadratic*
AID Ca	62.19 ^a^	47.93 ^b^	61.0 ^a^	61.91 ^a^	62.71 ^a^	0.779	*<0.001*	*<0.001*	*<0.001*
AID P	69.34 ^a^	57.93 ^b^	68.1 ^a^	69.82 ^a^	70.66 ^a^	0.704	*<0.001*	*<0.001*	*<0.001*

^a,b^ Means in the same row with no common superscripts are significantly different at a probability level of *p* < 0.05. SEM, pooled standard error of the mean.

**Table 8:** Economic analysis of full matrix application of a novel consensus phytase variant, on economic benefit.

	Day 1–35 Feed Cost/BWG (USD/kg)	Day 1–42 Feed Cost/BWG (USD/kg)
PC	0.458 ^a^	0.501
NC+PhyG500	0.452 ^ab^	0.486
NC+PhyG1000	0.442 ^bc^	0.478
NC+PhyG2000	0.438 ^c^	0.472
*p*-value	0.0006	0.136

^a–c^ Means in the same row with no common superscripts are significantly different at a probability level of *p* < 0.05.

## Data Availability

Data are unavailable due to privacy or ethical restrictions.
